# A nomogram prediction of overall survival based on lymph node ratio, AJCC 8th staging system, and other factors for primary pancreatic cancer

**DOI:** 10.1371/journal.pone.0249911

**Published:** 2021-05-05

**Authors:** Rui Zhong, Xin Jiang, Yan Peng, Huan Xu, Yongfeng Yan, Li Liu, Xiaowei Tang

**Affiliations:** 1 Department of Gastroenterology, Affiliated Hospital of Southwest Medical University, Luzhou, Sichuan, China; 2 Department of Digestive Endoscopy, The First Affiliated Hospital with Nanjing Medical University, Nanjing, China; 3 Department of General Surgery, The First Affiliated Hospital with Nanjing Medical University, Nanjing, China; University of Nebraska Medical Center, UNITED STATES

## Abstract

**Background:**

As a malignant tumor with poor prognosis, accurate and effective prediction of the prognosis of pancreatic cancer (PC) is crucial.

**Methods:**

A total of 12,909 patients diagnosed with pancreatic cancer were selected from the Surveillance, Epidemiology, and End Results program between 2004 and 2016. The sex, age, ethnicity, marital status, metastasis status, radiotherapy, chemotherapy, tumor size, regional nodes examined, regional nodes positive of each patient were recorded. Univariate and multivariate Cox regression analyses were used to identify prognostic factors with a threshold of P<0.05, and a nomogram was constructed. Harrell’s concordance indexes and calibration plots were used to verify the predictive power of the model. The risk groups were also stratified by quartile of the total score. Survival rates were estimated by the Kaplan-Meier method.

**Results:**

Age, year of diagnosis, sex, grade, histologic, marital, TNM stage, surgery of the primary site, tumor size, regional nodes positive and regional nodes examined ratio (LNR), lymph node dissection, radiotherapy, and chemotherapy were identified as prognostic factors for the construction of the nomogram. The nomogram exhibited a clinical predictive ability of 0.675(95% CI, 0.669~0.681) in the internal verification. The predicted calibration curve was similar to the standard curve. Decision curve analysis showed that the nomogram had value in terms of clinical application. Besides, the nomogram was able to divide the patients into different groups according to total points.

**Conclusions:**

Hence, our nomogram was highly effective in predicting overall survival in patients with PC, which may provide a reference tool for clinicians to guide individualized treatment and follow-ups for patients with PC, accurately determine the 1-,3- and 5-year overall survival of patients.

## Introduction

Pancreatic cancer (PC) is the seventh leading cause of cancer-related deaths worldwide with 432,242 related deaths in 2018 [1]. Surgical resection remains the only potentially curative therapy for patients with pancreatic cancer but offered to only 10% to 15% of patients with PC. Overall 5‐year survival after surgery is only 15% to 20% [2]. Several factors are related to the prognostic outcome of patients with PC, including TNM stage, histologic differentiation, tumor size, lymph node (LN) status, and age et. al [3]. A constructed nomogram using these parameters could aid in predicting malignant potential in patients with PC, however, there is no nomogram to combine these parameters using the eighth edition of AJCC.

## Materials and methods

### Data collection

A total of 12,909 patients with PC diagnosed between January 1, 2004, and December 31, 2016, were selected from the SEER database (https://seer.cancer.gov) by SEER*Stat software (version 8.3.8; Client-server: ssp://seerstat.imsweb.com:2038), and the incidence SEER 18 Regs Custom Data (with additional treatment fields), Nov 2018 Sub (1975–2016 varying), were selected for analysis. The inclusion codes and criteria from the SEER database were are as follows: The primary tumor site was coded as pancreas (C25.0-C25.9), the coding of tumor pathological tissue classification was adenocarcinomas (8140), infiltrating duct carcinoma (8500), and other (not including the pancreatic neuroendocrine tumors). The following parameters were collected: 1) Marital status; 2) ethnicity; 3) sex; 4) age at diagnosis; 5) survival time (months); 6) overall survival (OS) and cancer-specific survival (CSS); 7) regional nodes positive and examined; 8) lymph node dissection; 9) surgery of the primary site; 10) radiation therapy and chemotherapy received; 11) whether there was bone (not including the bone marrow), brain (not including the spinal cord or other parts of the central nervous system), lung (not including the pleura or pleural fluid) or liver metastasis; 12) tumor size(mm); 13) CS extension; 14) year of diagnosis. According to the SEER program definition, survival time means the time between diagnosis and death or the last follow-up time. OS is the time from the date of diagnosis to the death of any cause. CSS is the time from the date of diagnosis to the date of cancer-associated mortality. The TNM stage of the AJCC 8th edition was evaluated based on the following codes: bone metastasis, brain metastasis, lung metastasis, liver metastasis, Mets at DX-Distant LN (2016+), Mets at DX-Other (2016+), collaborative stage (CS) tumor size 2004–2015, CS extension 2004–2015, CS lymph nodes 2004–2015, CS metastases at DX 2004–2015, Regional nodes positive and derived AJCC stage group (7th edition; 2010+). According to the definition of the 8th Edition of the American Joint Committee on Cancer (AJCC) staging system [4], all the included patients with pancreatic cancer were stage IA-IV.

After obtaining the data, we set the exclusion criteria as follows: (1) patients whose clinical information were unknown or incomplete; (2) patients whose tumors were not diagnosed pathologically; (3) patients who were younger than 18 years of age; (4) patients whose survival time was less than one month; (5) patients with multiple primary cancers; (5) patients whose pancreatic neuroendocrine tumors (8013, 8041, 8150, 8151, 8152, 8153, 8154, 8155, 8156, 8240, 8240, 8241, 8242, 8243, 8244,8245/3, 8246, 8246, and 8249).

The SEER database does not include any human or demographic identifying information, and the data used for analysis were de-identified. Therefore, ethics approval and formal informed consent to participate was not required.

### Statistical analysis

Statistical operations were performed using SPSS 26.0 (IBM Corporation, Armonk, NY, USA) and constructed a nomogram using R software version 3.5.2 (https://www.r-project.org). The best cutoff value of LNR, age, tumor size, was determined by receiver operating characteristic (ROC) curve. The univariate and multivariate analyses and hazard ratios (HRs) were used by Cox proportional hazards regression model to find its independent prognostic risks, and P < 0.05 was considered as statistically significant difference. These independent risk factors were used to construct a nomogram using R software, using the rms and survival packages (https://www.rdocumentation.org/pack-ages/survival/versions/2.42-3). The nomogram used 1-, 3- and 5-year overall survival (OS) as end points. In internal verification, bootstraps of 1,000 resamples were used for analysis, Harrell’s concordance indexes(C-indexes) and calibration curves were used to verify the predicted effect of the nomogram, the nomogram and 8^th^ TNM staging systems were compared using decision curves analysis. Patients in the validation set were assigned into four groups according to the quartiles of their prognostic scores. The Kaplan-Meier method was used to estimate overall survival rate in the four groups, and the differences were evaluated using the log-rank test with a threshold of P<0.05.

## Results

### Baseline characteristics

The mean age of the patients was 65.7 years. The median OS was 17 months (range, 9–32 months). In addition, the 1-, 3- and 5-year OS rates were 65.7%, 27.2% and 17.8%, respectively. According to the TNM staging system of the AJCC 8th edition, the separate stage of patients was recorded. The basic information of the patients was presented in Table 1.

**Table 1:** Patient demographics (N = 12909).

Characteristic	N(%)
Age	65.7±10.8
Race	
White	10512 (81.4)
Black	1320 (10.2)
Other	1077 (8.3)
Sex, male	6552 (50.7)
Year of diagnosis	
2004~2010	6869 (53.2)
2011~2016	6040 (46.7)
Primary site	
Head of pancreas	9505 (73.6)
Body of pancreas	869 (6.7)
Tail of pancreas	1262 (9.7)
Other	1273 (9.8)
Histologic type	
Adenocarcinoma	6488 (50.2)
Infiltrating duct carcinoma	4923 (38.1)
Other	1498 11.6)
AJCC Stage Group,8th	
IA	933 (7.2)
IB	2039 (15.7)
IIA	918 (7.1)
IIB	4777 (37.0)
III	3371 (26.1)
IV	871 (6.7)
Surgery of the primary site	12263 (94.9)
Regional nodes examined	14 (8~21)
Regional nodes positive	1 (0~3)
Lymph node dissection	
No	574 (4.4)
1~3	996 (7.7)
≥4	11339 (87.8)
Radiation therapy	4473 (34.6)
Chemotherapy	8838 (68.4)
Grade	
Well differentiated(I)	1489 (11.5)
Moderately differentiated(II)	6417 (49.7)
Poorly differentiated(III)	4773 (36.9)
Undifferentiated(IV)	230 (1.7)
Marital status	
Unmarried	1938 (15.0)
Married	8202 (63.5)
Divorced	1286 (9.9)
Widowed	1483 (11.4)
Tumor size, mm	33 (25~44)
Dead (attributable to this cancer)	9374 (72.6)
Dead	10105 (78.2)
Survival time, months	17 (9~32)

### Cox regression analysis

The following factors were included in the univariate Cox regression analysis: age (<70 vs. ≥70), tumor size (<35mm vs. ≥35mm), LNR (<0.133 vs. ≥0.133), year of diagnosis (2004~2010 vs. 2011~2016), race (white vs. black vs. other), sex (male vs. female), marital status (married vs. unmarried vs. divorced vs. widowed), histological type (adenocarcinoma vs. infiltrating duct carcinoma vs. other), grade (well differentiated, grade I vs. moderately differentiated, grade II vs. poorly differentiated, grade III vs. undifferentiated or anaplastic, grade IV), primary site (head of pancreas vs. body of pancreas vs. tail of pancreas vs. other), TNM stage (IA vs. IB vs. IIA vs. IIB vs. III vs. IV), surgery at primary site (no vs. yes), scope of regional lymph node surgery (none vs. 1–3 regional lymph nodes removed vs. ≥4 regional lymph nodes removed), radiation therapy (no vs. yes) and chemotherapy (no vs. yes). The remaining prognostic factors with P<0.05 were included in the multivariate Cox regression analysis. The results demonstrated that residual factors, with the exception of the primary site and race were independent prognostic factors and were thus included in the construction of the nomogram (Table 2).

**Table 2:** Univariate and multivariate Cox regression analysis for OS in pancreatic cancer patients.

Variable	Univariate analysis	P-value	Multivariate analysis	P-value
	HR(95%CI)		HR(95%CI)	
Age at diagnosis				
<70	reference		reference	
≥70	1.263 (1.213~1.314)	<0.001	1.201 (1.152~1.253)	<0.001
Year of diagnosis				
2004~2010	reference		reference	
2011~2016	0.858 (0.824~0.894)	<0.001	0.881 (0.846~0.919)	<0.001
Sex				
Male	reference		reference	
Female	0.939 (0.903~0.976)	0.001	0.929 (0.892~0.968)	<0.001
Tumor size, mm				
<35	reference		reference	
≥35	1.332 (1.281~1.385)	<0.001	1.213 (1.162~1.267)	<0.001
Surgery of the primary site				
No	reference		reference	
Yes	0.393 (0.361~0.426)	<0.001	0.554 (0.491~0.624)	<0.001
Lymph node dissection		<0.001		<0.001
No	reference		reference	
1~3	0.610 (0.546~0.681)	<0.001	0.984 (0.868~1.117)	0.807
≥4	0.519 (0.474~0.568)	<0.001	0.843 (0.744~0.955)	0.007
LNR				
<0.133	reference		reference	
≥0.133	1.742 (1.675~1.812)	<0.001	1.287 (1.223~1.355)	<0.001
Radiation therapy				
No	reference		reference	
Yes	0.792 (0.760~0.825)	0.001	0.897 (0.857~0.939)	<0.001
Chemotherapy				
No	reference		reference	
Yes	0.721 (0.691~0.751)	<0.001	0.634 (0.605~0.665)	<0.001
Marital status		<0.001		<0.001
Unmarried	reference		reference	
Married	0.986 (0.932~1.044)	0.633	0.940 (0.888~0.995)	0.034
Divorced	1.062 (0.980~1.150)	<0.143	1.059 (0.977~1.147)	0.162
Widowed	1.199 (1.112~1.293)	<0.001	1.076 (0.994~1.165)	0.071
Race		0.087		
White	reference			
Black	1.007 (0.944~1.074)	0.832		
Other	0.922 (0.858~0.992)	0.030		
Primary site		<0.001		0.079
Head of pancreas	reference		reference	
Body of pancreas	0.892 (0.822~0.967)	0.006	0.989 (0.911~1.073)	0.784
Tail of pancreas	0.861 (0.804~0.922)	<0.001	0.916 (0.853~0.983)	0.015
Other	1.020 (0.955~1.090)	0.558	1.021 (0.955~1.092)	0.544
Histologic type		<0.001		<0.001
Adenocarcinoma	reference		reference	
Infiltrating duct	0.957 (0.918~0.997)	0.036	1.020 (0.977~1.064)	0.368
Other	0.642 (0.600~0.688)	<0.001	0.631 (0.587~0.678)	<0.001
AJCC Stage Group,8th		<0.001		<0.001
IA	reference		reference	
IB	1.501 (1.357~1.660)	<0.001	1.409 (1.272~1.561)	<0.001
IIA	1.554 (1.383~1.746)	<0.001	1.395 (1.231~1.580)	<0.001
IIB	2.201 (2.007~2.414)	<0.001	1.850 (1.676~2.043)	<0.001
III	2.890 (2.630~3.176)	<0.001	2.186 (1.962~2.436)	<0.001
IV	4.528 (4.048~5.065)	<0.001	3.249 (2.874~3.672)	<0.001
Grade		<0.001		<0.001
I	reference		reference	
II	1.611 (1.502~1.729)	<0.001	1.519 (1.414~1.632)	<0.001
III	2.179 (2.028~2.342)	<0.001	2.016 (1.873~2.169)	<0.001
IV	1.896 (1.615~2.225)	<0.001	2.299 (1.955~2.703)	<0.001

### Construction and validation of the nomogram

**Fig 1:**
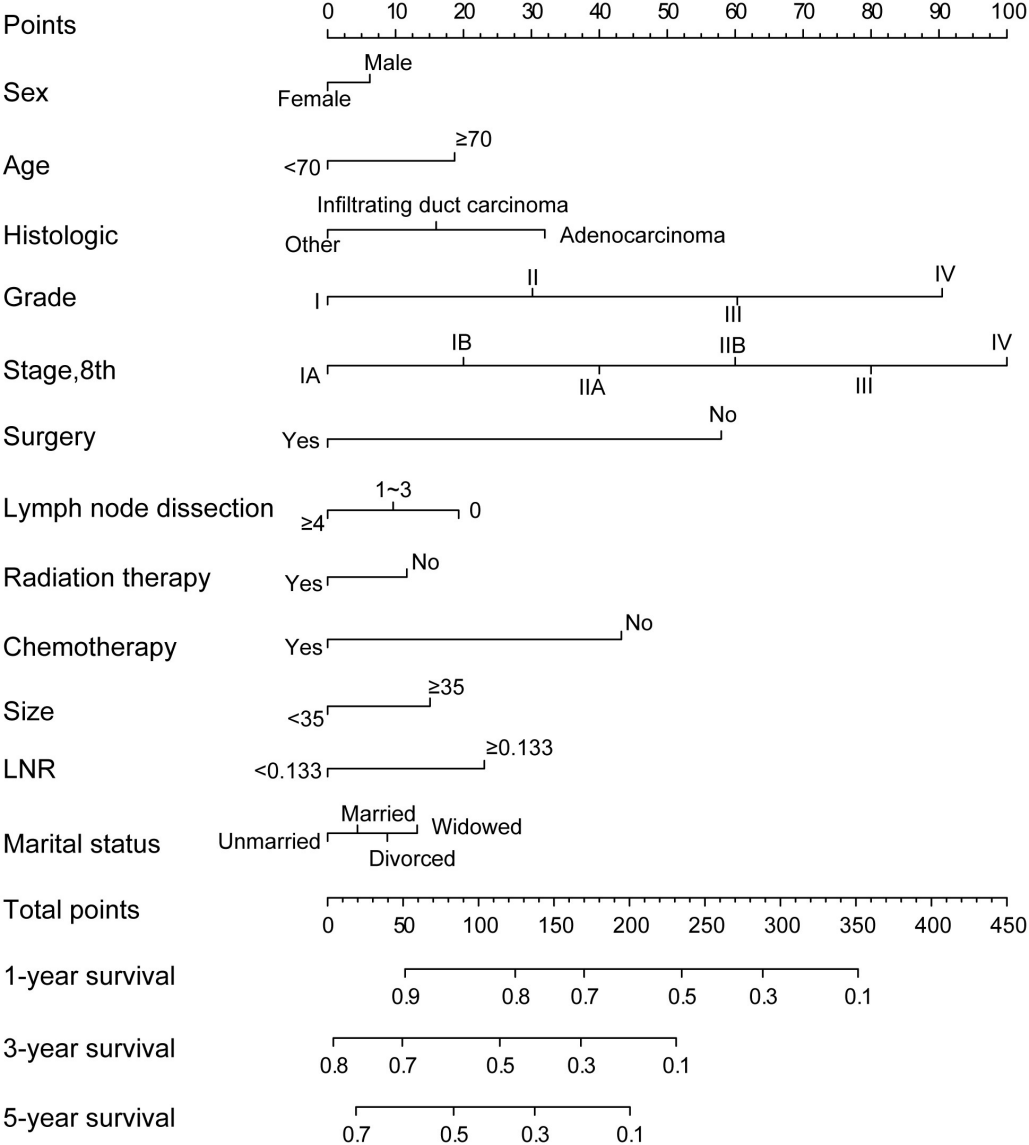
Nomogram for predicting the 1,3- and 5-year overall survival of patients with pancreatic cancer.

**Fig 2:**
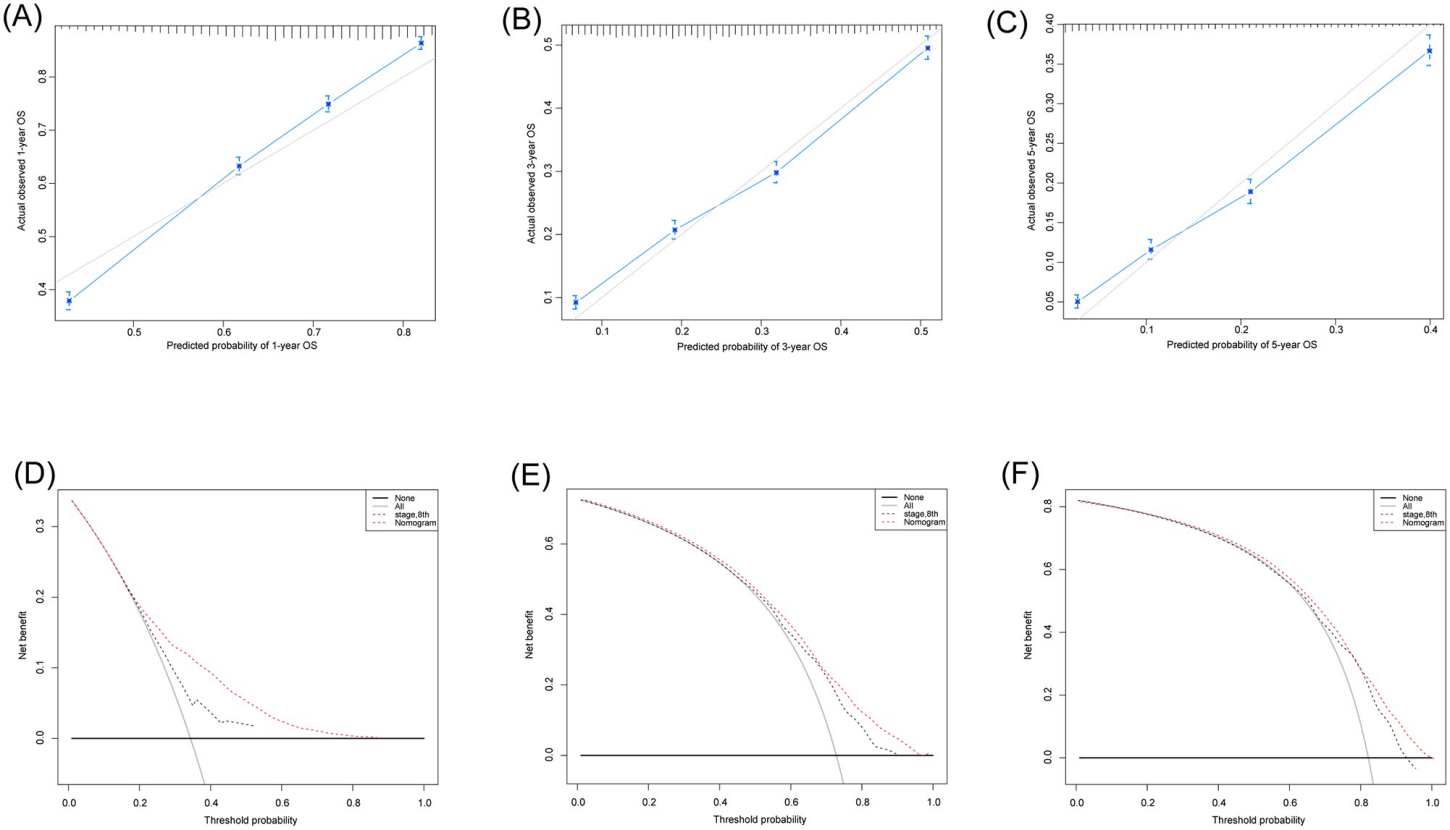
Calibration curves of the nomogram for predicting the 1- (A), 3- (B), and 5- (C) year OS rates of patients with PC. Decision curve analysis of the AICC 8th TNM stage, and nomogram for the 1- (D), 3- (E), and 5- (F) year OS rates of patients with PC. The black line represents the AICC 8th TNM stage, the red line represents the nomogram. OS: Overall survival; PC: Pancreatic cancer.

Marriage, sex, and year of diagnosis had a relatively small effect. The nomogram comprised 12 prognostic factors: age, sex, histologic, marital, grade, TNM stage, surgery, extent of lymph node dissection, LNR, tumor size, radiation therapy and chemotherapy. Surgery, TNM stage and grade exhibited the strongest impact on prognosis among all factors, chemotherapy also served an important role (Fig 1). The effects of other factors on prognosis were moderate. A total score was calculated by adding up the scores of each factor according to the different characteristics. The 1,3- and 5-year survival rates were estimated by drawing a straight line from the total score on the nomogram. The C-index calculated by the bootstrap self-sampling method was 0.675(95% CI, 0.669~0.681), indicating good predictability of the nomogram. In addition, the calibration curve was similar to the standard curve in predicting the 1, 3- and 5-year survival rates of patients, indicating good predictive ability of the nomogram (Fig 2).

### Decision curve analysis

After determining the accuracy and discriminative ability of the model, we performed comparison between the nomogram and 8^th^ TNM staging systems through the Decision Curve Analysis (DCA). The results showed that the nomogram had a good clinical applicability in predicting the survival of PC because of its wide range of threshold probabilities. In addition, the nomogram had an advantage over traditional 8^th^ TNM staging systems in predicting OS because the net benefit was higher (Fig 2).

### Risk stratification

**Fig 3:**
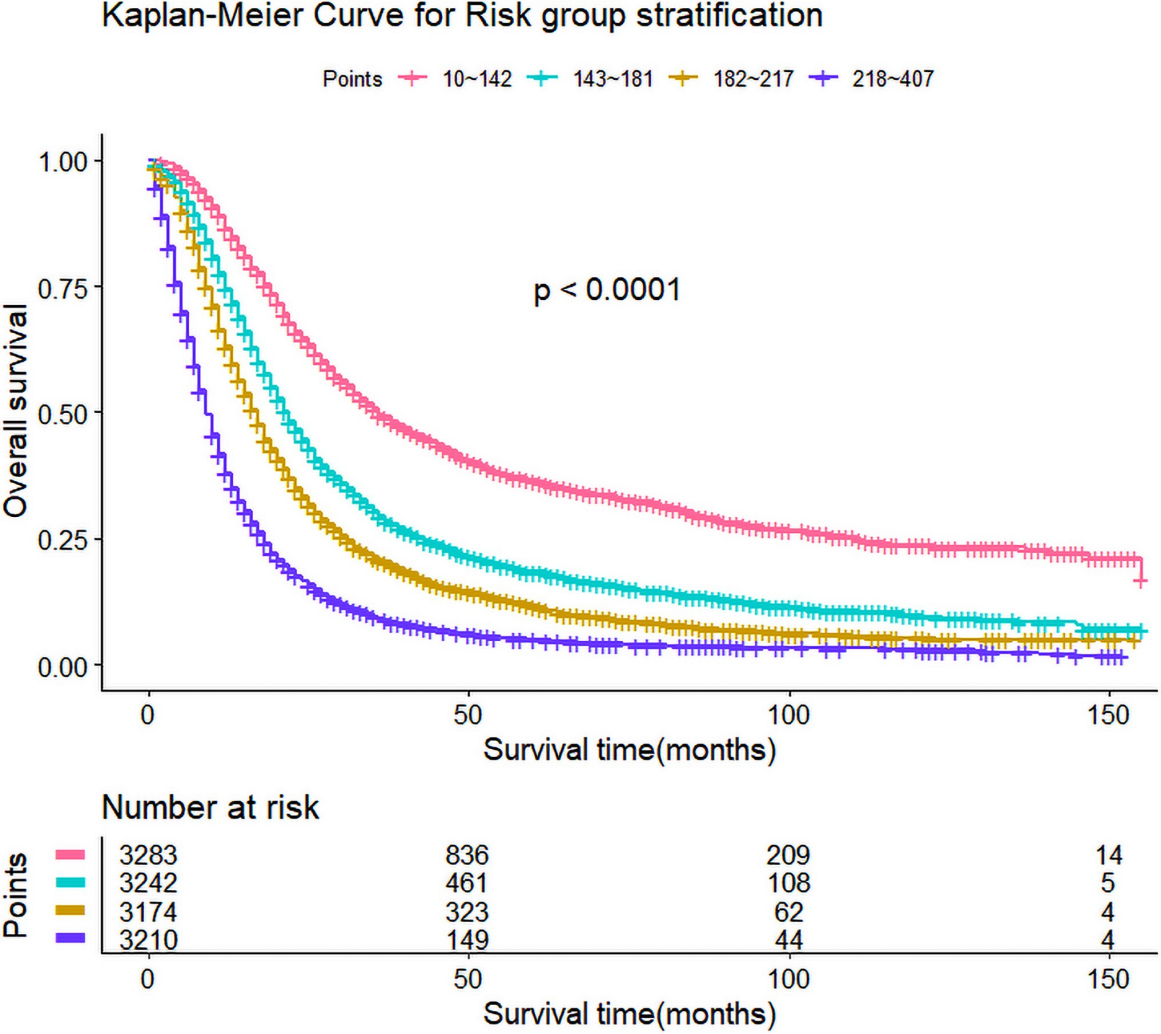
Risk group stratification among all patients.

The total score for each patient was calculated, and the scores divided into four subgroups through quartiles (10–142, 143–181, 182–217 and 218–407) to represent different outcomes. There was a significant difference of OS among the subgroups (Table 3; Fig 3).

**Table 3:** Scores of every subgroup within each variable in the nomogram.

Variable	Points	Variable	Points
Surgery of the primary site		AJCC Stage Group,8th	
No	58	IA	0
Yes	0	IB	20
Tumor size, mm		IIA	40
<35	0	IIB	60
≥35	15	III	80
Grade		IV	100
I	0	Chemotherapy	
II	30	No	43
III	60	Yes	0
IV	90	Marital status	
LNR		Unmarried	0
<0.133	0	Married	4
≥0.133	23	Divorced	9
Radiation therapy		Widowed	13
No	12	Age at diagnosis	
Yes	0	<70	0
Histologic type		≥70	19
Adenocarcinoma	32	Sex	
Infiltrating duct	16	Male	6
Other	0	Female	0
Lymph node dissection			
No	19		
1~3	10		
≥4	0		

## Discussion

To the best of our knowledge, this is the first large-scale clinical retrospective study that used the SEER database to construct a nomogram to predict survival rates for patients with PC based on lymph node ratio, AJCC 8th staging system. In this study, a total of 12,909 patients were included following rigorous screening, and 12 risk factors that significantly affected prognosis were determined by the Cox regression method. A nomogram was constructed based on these 12 risk factors. The C-index and the graphical calibration method were used for internal validation, which suggested that the nomogram exhibited a good predictive ability.

The nomogram demonstrated that the survival of patients with PC was affected by multiple factors, especially the treatment strategy. The nomogram also accurately predicted the prognosis of different risky groups. Compared with traditional 8th TNM staging, the model established in the present study combined more clinical information to determine the prognosis of patients more accurately and guide the future treatment strategy. We found surgery exhibited the strongest impact on prognosis, age and tumor size had a moderate influence on prognosis, and sex and marital status only had a minor effect. The demand and role of radiation therapy remained small, however, radiation therapy might have some importance as a local treatment [5]. In elderly patients with PC, the aging of organs coupled with a decrease in immune function leads to a high possibility of tumor recurrence. Elderly patients with PC exhibit low tolerance to surgery, radiotherapy and chemotherapy, and therefore, their compliance to anticancer treatment may be poor. Additionally, elderly patients who often suffer from other conditions and financial burden also needs to be considered, and thus their survival rate is reduced [6]. Thus, age is associated with the prognosis of patients with PC. In addition, Our multivariate regression analysis showed that the year of diagnosis was also a prognostic factor. It was indicative that the field has made progress in terms of treating patients with pancreatic cancer. Over the period selected for this study (2004 to 2016), there have been several changes in PC treatment. These changes include the addition of chemotherapy regimens that have increased efficacy compared to traditional Gemcitabine monotherapy (introduced in 1997) [7]. Similarly, the surgical treatment of PC has been augmented by the rise in the utilization of adjuvant and neoadjuvant chemotherapies.

The 8th edition of the AJCC staging system was released on October, 2016, and has been recommended to replace the old version in 2018 [8]. The new staging system for PC has notable modifications from the 7th edition, including new definitions for T and N classifications. The new T-staging system refers to tumor size without considering extra pancreatic invasion. Additionally, positive regional nodal involvement (previous N1) has been subdivided into N1 (1–3 positive regional lymph nodes) and N2 (≥ 4 positive regional lymph nodes). These changes are consequential, and many studies have investigated the validity of the 8th AJCC staging system for PC [4,9], Our results also show that the eighth edition staging dominates the nomogram score. To further understand the differences between the 7th and the 8th editions of the staging system, Fudan University Shanghai Cancer center has simultaneously used both systems for PC patients since 2017, they have not found the new staging system to be as accurate as expected. Therefore, they proposed some improvements on the 8th edition of the staging system [10].

Tumor grade is a measure of the degree of differentiation of the tumor. Roughly, it measures how closely the malignant cells resemble the morphologic and functional characteristics of the tissue of origin. In addition, tumor differentiation reflected the biological behaviors of PC, which was highlighted in several studies for its vital role in survival. This is likely because less differentiated tumors possess a more aggressive biology, leading to earlier local and distant metastasis [11]. Tumor grade has already been accepted as part of the AJCC staging system for prostate cancer and sarcoma on the basis of the ability to discriminate differences in overall survival within those diseases [12]. Our analysis has demonstrated that tumor grade in PC is capable of doing the same and should therefore be strongly considered for addition to the AJCC staging system.

LNR was confirmed as an independent prognostic risk factor in the univariate and multivariate analyses, as well as age, grade, and T classification. As it was well known, lymph node involvement appeared to be one of the most important risk for predicating OS of resected PC patients [13]. Nevertheless, the total number of examined positive lymph nodes was still imperfect as a pivotal predictor owing to its influence on surgical procedures. Ning Pu et.al. found that LNR did not appear to be associated with surgical procedures, because no matter how expansive of lymph nodes surgery was, LNR reflected its ability in involvement and metastasis, while absolute positive lymph node counts was severely affected by the scope [14]. Extended lymphadenectomy may not be necessary for PC patients, because it could increase the postoperative complications, morbidities, and mortalities, and even influence the quality of life [15,16].

In general, the assessment of LNR could make patients utmostly benefit from the surgery and still be evaluated accurately on their survival risks, which may guide the clinical treatment. As large amounts of factors turned up as prognostic indicators for resected PC patients [17–19], the AJCC 8th staging system seemed to lose its powerful efficiency in the evaluation of prognosis. Nomogram, a quantitative rating predictive model, had revealed its mighty power in survival prediction, which may have the chance to replace the TNM staging system. Compared to the AJCC 8th staging system, the concept of tumor size and lymph nodes status were still involving in the formulated nomogram. Besides this, age and differentiation grade were further incorporated into the novel model. Asano et.al [20] had reported the role of age in the survival of resected PC patients. In addition, tumor differentiation reflected the biological behaviors of PC, which was highlighted in several studies for its vital role in survival [21]. Thus, the formulated nomogram merged T, N, M status and other significant factors together to obtain the much more precise model specially with the validation of superior consistent calibration curves and wider ranges of DCA.

The present study had several limitations. First, due to the limited information available in the SEER database, drinking history, radiotherapy dose, specific chemotherapy regimen, surgical methods and additional clinical information could not be obtained, which may have affected the results. Second, the SEER database provide the TNM staging was based on the 7th edition of the AJCC staging system, according to the size of the tumor, the degree of tumor infiltration (involving the abdominal cavity, superior mesenteric artery, etc.), regional lymph node metastasis, and distant metastasis, all stage were transferred to the 8th edition. In this process, we deleted many missing fields, resulting in a relatively small number of patients with stage IV, which may affect our final results. Third, the patients with PC in the SEER database were all from the United States, and although patients of different races were included, the cohort may not be representative of patients worldwide. Finally, the study design was retrospective essentially, so a large-scale and multicenter prospective study should be launched to verify our results and eliminate the selective bias. Next, the cutoff value of all continuous variables used in our study may not be appropriate to other studies, and a meta-analysis may be required to determine the most suitable cutoff value.

## Conclusion

The proposed nomogram containing TNM classification, LNR, age, tumor size, and grade reveals a superior prognostic model. In addition, the formulated nomogram staging system confirmed its excellent discrimination and risk stratification compared to the AJCC 8th staging system.

## Supporting information

S1 Fig Survival curves. Survival curve of (A) sex, (B) year of diagnosis, (C) age, (D) histologic, (E) grade, (F) stage, (G) surgery, (H) lymph node dissection, (I) Radiation therapy, (J) Chemotherapy, (K) size,(L) LNR, (M) Marital status. These graphs show the impact of each subtype on survival.
(TIF)

S1 Table Correlations between LNR and characteristics of patients with PC. (DOCX)
