# Supplementary figures and images for: A nomogram prediction of overall survival based on lymph node ratio, AJCC 8th staging system, and other factors for primary pancreatic cancer
Source: PLoS One. 2021 May 5;16(5):e0249911. doi: 10.1371/journal.pone.0249911 (PMC8099056; doi:10.1371/journal.pone.0249911)

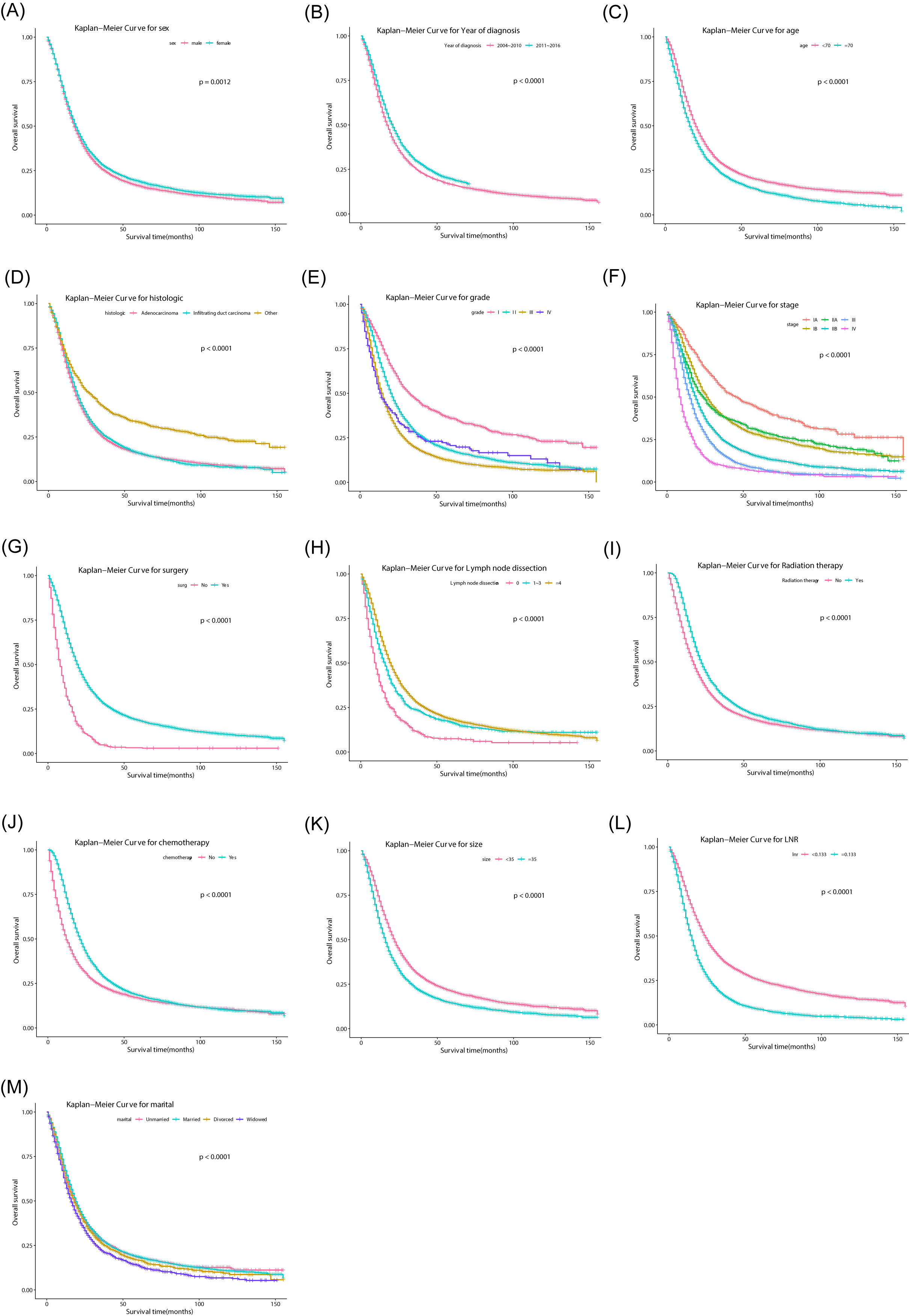

Supplement: S1 Fig — Survival curve of (A) sex, (B) year of diagnosis, (C) age, (D) histologic, (E) grade, (F) stage, (G) surgery, (H) lymph node dissection, (I) Radiation therapy, (J) Chemotherapy, (K) size,(L) LNR, (M) Marital status. These graphs show the impact of each subtype on survival. (TIF) [file pone.0249911.s001.tif]
