# Supplementary material for: A nomogram prediction of overall survival based on lymph node ratio, AJCC 8th staging system, and other factors for primary pancreatic cancer
Source: PLoS One. 2021 May 5;16(5):e0249911. doi: 10.1371/journal.pone.0249911 (PMC8099056; doi:10.1371/journal.pone.0249911)
Supplement: S1 Table — (DOCX) [file pone.0249911.s002.docx]

**S1 Table.** **Correlations between LNR and characteristics of patients with PC.**

| **Variables** | **LNR<0.133** | **LNR≥0.133** | **P-value** |
| --- | --- | --- | --- |
| Age at diagnosis |  |  | 0.014 |
| <70 | 4409(55.3) | 3565(44.7) |  |
| ≥70 | 2838(57.5) | 2097(42.5) |  |
| Year of diagnosis |  |  | 0.003 |
| 2004~2010 | 3773(54.9) | 3096(45.1) |  |
| 2011~2016 | 3474(57.5) | 2566(42.5) |  |
| Sex |  |  | <0.001 |
| Male | 3565(54.4) | 2987(45.6) |  |
| Female | 3682(57.9) | 2675(42.1) |  |
| Tumor size, mm |  |  | <0.001 |
| <35 | 4064(60.4) | 2662(39.6) |  |
| ≥35 | 3183(51.5) | 3000(48.5) |  |
| Surgery of the primary site |  |  | <0.001 |
| No | 295(45.7) | 351(54.3) |  |
| Yes | 6952(56.7) | 5311(43.3) |  |
| Lymph node dissection |  |  | <0.001 |
| No | 258(44.9) | 316(55.1) |  |
| 1~3 | 624(62.7) | 372(37.2) |  |
| ≥4 | 6365(56.1) | 4974(43.9) |  |
| Radiation therapy |  |  | 0.108 |
| No | 4779(56.7) | 3657(43.3) |  |
| Yes | 2468(55.2) | 2005(44.8) |  |
| Chemotherapy |  |  | <0.001 |
| No | 2415(59.3) | 1656(40.7) |  |
| Yes | 4832(54.7) | 4006(45.3) |  |
| Marital status |  |  | 0.016 |
| Unmarried | 1083(55.9) | 855(44.1) |  |
| Married | 4550(55.5) | 3652(44.5) |  |
| Divorced | 725(56.4) | 561(43.6) |  |
| Widowed | 889(59.9) | 594(40.1) |  |
| Race |  |  | <0.001 |
| White | 5819(55.4) | 4693(44.6) |  |
| Black | 771(58.4) | 549(41.6) |  |
| Other | 657(61.0) | 420(39.0) |  |
| Primary site |  |  | <0.001 |
| Head of pancreas | 5062(53.3) | 4443(46.7) |  |
| Body of pancreas | 607(69.9) | 262(30.1) |  |
| Tail of pancreas | 798(63.2) | 464(36.8) |  |
| Other | 780(61.3) | 493(38.7) |  |
| Histologic type |  |  | <0.001 |
| Adenocarcinoma | 3558(54.8) | 2930(45.2) |  |
| Infiltrating duct | 2726(55.4) | 2197(44.6) |  |
| Other | 963(64.3) | 535(35.7) |  |
| AJCC Stage Group,8th |  |  | <0.001 |
| IA | 933(100) | 0(0) |  |
| IB | 2039(100) | 0(0) |  |
| IIA | 918(100) | 0(0) |  |
| IIB | 2520(52.8) | 2257(47.2) |  |
| III | 498(14.8) | 2873(85.2) |  |
| IV | 339(38.9) | 532(61.1) |  |
| Grade |  |  | <0.001 |
| I | 995(66.8) | 494(33.2) |  |
| II | 3658(57.0) | 2759(43.0) |  |
| III | 2460(51.5) | 2313(48.5) |  |
| IV | 134(58.3) | 96(41.7) |  |
